# Editorial: Neuroinflammation: mechanisms and therapeutic interventions

**DOI:** 10.3389/fimmu.2026.1815035

**Published:** 2026-03-24

**Authors:** Ma. Cecilia Opazo, Marcelo E. Ezquer, Mayra A. Machuca, Luisa F. Duarte

**Affiliations:** 1 Instituto de Ciencias Naturales, Facultad de Medicina Veterinaria y Agronomía, Universidad de Las Américas, Santiago, Providencia, Chile; 2 Centro de Investigación en Ciencias Biológicas y Químicas, Universidad de Las Américas, Santiago, Chile; 3 Centro de Medicina Regenerativa, Facultad de Medicina, Clínica Alemana – Universidad del Desarrollo, Santiago, Chile; 4 Escuela de Microbiología, Universidad Industrial de Santander, Bucaramanga, Colombia; 5 Millennium Institute on Immunology and Immunotherapy, Santiago, Chile

**Keywords:** glial activation, neurocognitive disorders, neurodegenerative diseases, neuroimmune crosstalk, neuroinflammation

Across the studies included in this Research Topic, neuroinflammation is no longer viewed as a simple or unidirectional process. Instead, it is increasingly understood as a complex and context-dependent network of biological responses, that involve multiple glial states, signals from peripheral immune system, metabolic and oxidative conditions, and different disease stages.

Moreover, the field is moving away from the idea that individual inflammatory molecules, such as single cytokines, are solely responsible for disease processes. Rather, researchers are adopting a systems-level perspective, that examine how multiple pathways interact to shape the overall inflammatory response and how interventions targeting these elements may modulate and mitigate neuroinflammation.

This shift reflects the understanding that neuroinflammation operates through interconnected regulatory networks rather than isolated signaling events. Consequently, therapeutic strategies must consider not only the direct effects of targeting a particular molecule or signaling pathway, but also the possibility of collateral biological outcomes triggered by compensatory mechanisms or unintended effects elsewhere in the network.

A central framework for this topic is provided by the mini-review by Müller et al., which highlights the dual nature of neuroinflammation within the interconnected environment of the brain. Rather than being activated in a simple on-off manner, microglia and astrocytes undergo a series of regulatory transitions. These changes are orchestrated by complex cytokine signaling pathways, receptor-mediated interactions, metabolic signals from the cellular environment, and continuous feedback from neurons. This dynamic and multi-layered regulation illustrates that neuroinflammation is an adaptive network process shaped by different players within the brain.

In this view, inflammation is not merely an output of pathology, it is also an adaptive regulatory system, that contributes to tissue repair and neural resilience but becomes maladaptive when inflammatory resolution fails or when persistent stimuli, such as aging, protein aggregation or cellular stress lock glia cells into chronic proinflammatory states. The original research submissions included here provide practical examples that support and expand this evolving framework. Each study addresses a specific mechanism involved in neuroinflammatory regulation. These mechanisms include damage-associated molecular patterns (DAMPs)-signaling linked to redox balance, as reported by Ullah et al., mitochondrial quality control and transfer communication discussed in the review by Ma et al., the regulation of inflammasome stability through autophagy–lysosomal pathways, and microRNA-mediated control of immunometabolic neurotoxins, among others. Collectively, these findings underscore the importance of identifying and modulating critical nodes within the neuroinflammatory network, moving the field toward more accurate and effective therapeutic strategies.

A clear example of this multi-target approach is the study by Liu et al. examining a combination therapy of curcumin and *Glycyrrhiza glabra* in an Alzheimer’s disease (AD) model. Using a D-galactose/sodium nitrite-induced mouse model, the authors reported that curcumin alone modestly improved spatial learning and reduced IL-6 levels, while *Glycyrrhiza glabra* alone showed limited effects. In contrast, the combined treatment produced the most pronounced behavioral improvements and broader biochemical impact, including the reduction of proinflammatory cytokines and the increasement of antioxidant capacity. The authors suggest that modulation of TLR4/MyD88/NF-κB signaling and redox pathways may underlie these observed anti-inflammatory and antioxidant effects, although direct assessment of pathway activation remains to be performed.

Mechanistic precision is further illustrated in the Parkinson’s disease (PD) study on DJ-1, a multifunctional protein implicated in PD pathogenesis, and its role in microglial NOD-like receptor family pyrin domain-containing 3 (NLRP3) inflammasome activation. Miao et al. reported that inflammatory stimuli increase DJ-1 levels, whereas reduction of DJ-1 suppresses NLRP3 inflammasome signaling (but not NLRC4 or AIM2), as shown by decreased caspase-1 activity and interleukin-1β (IL-1β) production. DJ-1 binds to and stabilizes NLRP3, preventing its autophagic degradation, a mechanism confirmed using autophagy modulators. These findings suggest that modulation of DJ-1 in microglia may attenuate neuroinflammation and confer neuroprotective effects. It will also be important to assess whether targeting this interaction can preserve beneficial microglial functions, such as phagocytosis, while preventing harmful IL-1β-driven inflammatory responses. Addressing these points will help clarify how inflammasome-targeted strategies might promote tissue repair and prevent chronic neuroinflammation.

Kezai et al. extend the concept of precision targeting by linking microRNA control to neurotoxic immunometabolism. Their study highlights the role of miR-132/212 in regulating the kynurenine pathway (KP) during lipopolysaccharide (LPS)-induced neuroinflammation. The KP links inflammation with the production of neuroactive metabolites, such as quinolinic acid (QUIN), a compound known for its neurotoxic effects. Subtoxic LPS exposure increases the expression of KP enzymes, particularly kynurenine 3-monooxygenase (KMO), leading to elevated QUIN levels in the brain. In miR-132/212 knockout mice, KMO activity remains elevated even after the inflammatory stimulus resolves, indicating that miR-132/212 plays a crucial role in shutting down this pathway. Importantly, overexpression of miR-132 in microglial cells reduces KMO expression and QUIN production without impairing microglial immune functions. These findings suggest that targeting the KP through miR-132/212 may reduce neurotoxicity with preserving essential immune responses. The study also raises important questions regarding cell-specific KP regulation, optimal timing for intervention, and how miRNA-based strategies might complement other anti-inflammatory treatments, highlighting KMO as a promising therapeutic target.

Two reviews expand our understanding of inflammatory networks beyond traditional neurodegeneration. Zhang et al. describe how interactions between peripheral neutrophil extracellular traps (NETs) and microglia contribute to secondary brain injury following intracerebral hemorrhage. In this context, neutrophils rapidly release NETs that cause tissue damage, promote blood–brain barrier (BBB) disruption, and induce inflammation. Therapeutic strategies, such as peptidylarginine deiminase 4 (PAD4) inhibitors and DNases are focused on disrupting NETs structures, rather than just blocking cytokine signaling. On the other hand, Li et al. examine the pathophysiology of high-altitude cerebral edema, linking it with hypoxia, oxidative stress, mitochondrial dysfunction, BBB disruption, neuroinflammation, and apoptosis.

On the other hand, several studies included in this editorial highlight the essential role of glial cells and their interactions, along with key peripheral immune mechanisms as central regulators of both tissue damage and repair during viral infections. Garcia Diaz et al. investigated the mechanisms underlying neuroinflammatory disease associated with Zika virus (ZIKV) infection in immunocompetent adult mice infected via footpad injection. Their results revealed significant apoptosis in the hippocampus and cortex, early infiltration of immune cells, sustained microglial activation, and persistent activation of inflammatory pathways for up to 4 weeks after acute infection, demonstrating prolonged neuroinflammation in the brain alongside viral persistence. A key finding was the role of infiltrating monocytes in driving microglial activation, as blocking monocyte recruitment using a CCR2 inhibitor (RS-102895) attenuated inflammatory gene signatures. These results position the monocyte–microglia axis as a potential therapeutic target in ZIKV-associated neuropathogenesis.

In parallel, Zhao et al. synthesize evidence showing that inflammatory eye manifestations, including conjunctivitis, anterior uveitis, and chorioretinitis, may occur during or after SARS-CoV-2 infection. This review frames ocular involvement as more than a local symptom, discussing how immune responses and tissue susceptibility in ocular compartments could contribute to persistent or delayed disease presentations. By integrating clinical observations with mechanistic hypotheses, the authors outline priorities for further studies, biomarker development, and rational anti-inflammatory strategies that preserve necessary antiviral immunity while limiting chronic tissue injury.

Jiang et al. further underline S100A8/A9 protein as damage-associated molecular pattern (DAMP)-like amplifiers linking infection, immune signaling, chronic neuroinflammation, and chronic pain. These proteins are proposed as both biomarkers and potential therapeutic targets, as they may contribute to the development of acute zoster neuralgia and its progression toward postherpetic neuralgia by sustaining inflammatory signaling in neural tissues. Carbone-Schellman et al. complement this perspective with a review summarizing recent preclinical and clinical studies investigating the use of mesenchymal stem cell (MSCs) in the context of viral infections in the central nervous system. Their work underscores the immunomodulatory and neuroprotective properties of MSCs and discusses their potential to modulate neuroinflammatory responses in infectious neurological diseases.

Regarding ischemic stroke (IS), Xiao et al. provide an integrated perspective on the complexity of post-ischemic inflammation and analyze the role of inflammatory markers in diagnosis, prognosis, and treatment. Their review also highlights advances in both conventional therapeutic strategies and traditional Chinese medicine, proposing integrated approaches that may improve clinical management.

Similarly, Li et al. focus on a major yet often underrecognized consequence of cerebrovascular disease: glial pyroptosis. They describe how the NLRP3/Caspase-1/Gasdermin D (GSDMD) axis acts as a mechanistic bridge linking ischemia-driven mitochondrial dysfunction and reactive oxygen species (ROS) production with sustained pro-inflammatory cytokine release. This process contributes to BBB disruption, neuronal injury, and impaired synaptic plasticity.

Another experimental evidence is provided by Yang et al., who investigated the effects of coixol in an 1-methyl-4-phenyl-1,2,3,6-tetrahydropyridine (MPTP)-induced PD mouse model. Their findings showed that coixol reduces neuroinflammation through inhibition of the nuclear transcription factor κB (NF-κB) and the mitogen-activated protein kinase (MAPK) signaling pathways, while also suppressing NLRP3 inflammasome activation and protecting mitochondrial function. These results support the idea that interventions targeting both inflammatory cascades and mitochondrial stress responses may produce amplified neuroprotective effects.

Moreover, Ju and Hang analyze neuroinflammation following intracerebral hemorrhage (ICH), elucidating its interaction with brain iron metabolism. This review integrates multiple pathological processes, including temporal stages of neuroinflammation, dysregulated iron homeostasis, and key regulatory signaling pathways. This comprehensive analysis provides valuable insights into future therapeutic strategies aimed at reducing secondary brain injury after ICH.

In a complementary analysis, He et al. presented a detailed overview of the role of the NLRP3 inflammasome in secondary injury following ICH. The authors emphasize that NLRP3 activation in microglia and astrocytes can drive glia cells toward pro-inflammatory states, exacerbating tissue damage. They also highlight the importance of understanding the regulatory crosstalk between these cell types and discuss emerging inhibitor strategies while noting critical challenges related to timing and cell-specific targeting.

Extending these insights beyond infectious and cerebrovascular contexts, several studies underscores the broader role of glial signaling in shaping neurological damage and recovery. Liu et al. using single-nucleus transcriptomics of the human hippocampus, demonstrate that sepsis induces profound neurovascular dysfunction characterized by pro-inflammatory glial polarization, dysfunctional astrocyte-microglia communication, and transcriptional signatures associated with BBB disruption, providing a cellular explanation for persistent neuroinflammation and cognitive impairment.

Notably, Zhu et al. showed that repetitive transcranial magnetic stimulation alleviates neuropathic pain by promoting anti-inflammatory microglial polarization through modulation of the methyltransferase-like 3 (METTL3)/*N*-methyl-d-aspartate receptor subtype 2B (NMDAR2B)/NLRP3 signaling axis. Meanwhile, Hao et al. present experimental evidence linking spinal cord ischemia-reperfusion injury (SCIRI) to neuropathic pain through alterations in microglial polarization. Their work report that miR-148a-3p targets SMAD specific E3 ubiquitin protein ligase 2 (SMURF2), reducing Sirtuin 1 (SIRT1) ubiquitination and promoting an anti-inflammatory microglial phenotype. Notably, intrathecal administration of miR-148a-3p improves pain-related behaviors, supporting the concept of microglial “reprogramming” as a promising therapeutic approach.

Xu et al. examined immune responses following optic nerve injury and highlight the dual role of neuroinflammation showing how glial activation, adaptive immune involvement, and immune checkpoint signaling collectively determine the balance between neuroprotection and neurodegeneration. Mincheva et al. further demonstrated that dampening neuroinflammation can shift disease-relevant glial states in dopaminergic degeneration. Using a 6-hydroxydopamine hydrochloride (6-OHDA) model, they report that golexanolone reduces microglial activation and decreases pro-inflammatory mediators such as tumor necrosis factor alpha (TNF-α), IL-1-α, and high mobility group box 1 (HMGB1). Their findings also reveal reduced activation of pro-inflammatory A1 astrocytes, which reinforce the interconnected regulation of glial populations.

Furthermore, Sun et al. demonstrated that alpha-lipoic acid (LA) mitigates peripheral neuropathy induced by nanoparticle albumin-bound paclitaxel (nab-PTX), a widely used chemotherapeutic agent. Their findings implicate several signaling pathways, including IL-17 signaling, in the neuroprotective effects of LA, highlighting the therapeutic potential of targeted immunomodulation in neuroinflammatory conditions.

Ultimately, Szumutku et al. present a clinical case with direct translational implications. In a child with fulminant acute hemorrhagic leukoencephalitis (AHLE) and rapidly increasing intracranial pressure, early decompressive craniectomy combined with therapeutic plasma exchange resulted in full recovery after one year. The underlying mechanistic clue was complete complement factor I deficiency, positioning complement dysregulation as a key driver rather than a secondary finding. The authors propose that complement testing in severe neuroinflammatory syndromes of unclear etiology may guide life-saving therapeutic decisions.

We hope that this collection helps readers identify both mechanistic anchors and practical translational pathways that accelerate the transition from neuroimmune insight to therapeutic impact. Neuroinflammation should be understood as a multi-layered process, that integrate cellular phenotypes, signaling pathways, and metabolic states rather than relying on single biomarkers. Future therapeutic strategies should prioritize restoring physiological immune balance rather than broadly suppressing inflammation. Combination therapies targeting interconnected nodes within neuroinflammatory network may ultimately offer the most effective path toward promoting neurological health.

